# Supplementary material for: A distributed residue network permits conformational binding specificity in a conserved family of actin remodelers
Source: eLife. 2021 Dec 2;10:e70601. doi: 10.7554/eLife.70601 (PMC8639148; doi:10.7554/eLife.70601)
Supplement: Supplementary file 1. [file elife-70601-supp1.pdf]

Protein sequences for *A distributed residue network permits conformational binding specificity in a conserved family of actin remodelers*

Constructs below were cloned into a pMCSG7 backbone (gift from F. Gertler) which encodes an N-terminal 6xHis-TEV site. Amino acid sequences of proteins are as given:

ENAH EVH1
MHHHHHHSSGVDLGTENLYFQSNAMSEQSICQARAAVMVYDDANKKWVPAGGSTGF
SRVHIYHHTGNNTFRVVGRKIQDHQVVINCAIPKGLKYNQATQTFHQWRDARQVYGLN
FGSKEDANVFASAMMHALEVL*
VASP EVH1
MHHHHHHSSGVDLGTENLYFQSNAMSETVICSSRATVMLYDDGNKRWLPAGTGPQA
FSRVQIYHNPTANSFRVVGRKMQPDQQVVINCAIVRGVKYNQATPNFHQWRDARQV
WGLNFGSKEDAAQFAAGMASALEALE*
EVL EVH1
MHHHHHHSSGVDLGTENLYFQSNAMSEQSICQARASVMVYDDTSKKWVPI**K**PGQQG
FSRINIYHNTASSTFRVVGVKLQDQQVVINYSIVKGLKYNQATPTFHQWRDARQVYGL
NFASKEEATTFSNAMLFALNIMNSQE*
EVL EVH1 V65P#
MHHHHHHSSGVDLGTENLYFQSNAMSEQSICQARASVMVYDDTSKKWVPIPGQQGF
SRINIYHNTASNTFRVVGVKLQDQQVVINYSIPKGLKYNQATPTFHQWRDARQVYGLN
FASKEEATTFSNAMLFALNIM*
EVL EVH1 V65P Y62C#
MHHHHHHSSGVDLGTENLYFQSNAMSEQSICQARASVMVYDDTSKKWVPIPGQQGF
SRINIYHNTASNTFRVVGVKLQDQQVVINCSIPKGLKYNQATPTFHQWRDARQVYGLN
FASKEEATTFSNAMLFALNIM*
EVL EVH1 swapped (7-residue swapped)#
MHHHHHHSSGVDLGTENLYFQSNAMSEQSICQARASVMVYDDTSKKWVPAGGQQG
FSRINIYHNTASNTFRVVGVKLQDQQVVINCSIPKGLKYNQATPTFHQWRDARQVYGL
NFASKEEATTFANAMLFALEIL*
ENAH EVH1-PCARE (for crystallography)
MHHHHHHSSGVDLGTENLYFQSNAMSEQSICQARAAVMVYDDANKKWVPAGGSTGF
SRVHIYHHTGNNTFRVVGRKIQDHQVVINCAIPKGLKYNQATQTFHQWRDARQVYGLN
FGSKEDANVFASAMMHALEVLGGSGSGAAKSEELSCEMEGNLEHLPPPPMEVLMDK
SFASLES*
SUMO-ActA
MAGGLNDIFEAQKIEWHEDTGGSSHHHHHHGSGSGSDSEVNQEAKPEVKPEVKPET
HINLKVSDGSSEIFFKIKKTTPLRRLMEAFAKRQGKEMDSLTFLYDGIEIQADQTPEDLD
MEDNDIIEAHREQIGGGFNAPATSEPSSFEFPPPPTEDELEIIRETASSLDS*
SUMO-PCARE B
MAGGLNDIFEAQKIEWHEDTGGSSHHHHHHGSGSGSDSEVNQEAKPEVKPEVKPET
HINLKVSDGSSEIFFKIKKTTPLRRLMEAFAKRQGKEMDSLTFLYDGIEIQADQTPEDLD
MEDNDIIEAHREQIGGSGSGNLEH**LPPPP**MEVLMDKSFASLES

These EVL constructs had a single-residue deletion of WT EVL residue Lys 27, which was removed to make the lengths of ENAH EVH1 and the mutated EVL EVH1 domains

equal. In PDB structure 1QC6, Lys27 is at the end of a loop that is disordered and has a high B-factor.

Constructs below were cloned into a pCIB lentiviral expression vector for use in mammalian cell culture.

Mito-6xHis-mRuby2-PCAREB
MVGRNSAIAAGVCGALFIGYCIYFDRKRRSDPNFKSRMSRATMHHHHHHMVSKGEELI
KENMRMKVVMEGSVNGHQFKCTGEGEGNPYMGTQTMRIKVIEGGPLPFAFDILATSF
MYGSRTFIKYPKGIPDFFKQSFPEGFTWERVTRYEDGGVVTVMQDTSLEDGCLVYHV
QVRGVNFPSNGPVMQKKTKGWEPNTEMMYPADGGLRGYTHMALKVDGGGHLSCSF
VTTYRSKKTVGNIKMPGIHAVDHRLERLEESDNEMFVVQREHAVAKFAGLGGGMDEL
YKSGLRSQASAVDGTAGEMEGNLEHLPPPPMEVLMDKSFASLES*

6xHis-mRuby2-PCAREB
MHHHHHHMVSKGEELIKENMRMKVVMEGSVNGHQFKCTGEGEGNPYMGTQTMRIK
VIEGGPLPFAFDILATSFMYGSRTFIKYPKGIPDFFKQSFPEGFTWERVTRYEDGGVVT
VMQDTSLEDGCLVYHVQVRGVNFPSNGPVMQKKTKGWEPNTEMMYPADGGLRGYT
HMALKVDGGGHLSCSFVTTYRSKKTVGNIKMPGIHAVDHRLERLEESDNEMFVVQRE
HAVAKFAGLGGGMDELYKSGLRSQASAVDGTAGEMEGNLEHLPPPPMEVLMDKSFA
SLES*
