# Supplementary material for: A distributed residue network permits conformational binding specificity in a conserved family of actin remodelers
Source: eLife. 2021 Dec 2;10:e70601. doi: 10.7554/eLife.70601 (PMC8639148; doi:10.7554/eLife.70601)
Supplement: Supplementary file 2. [file elife-70601-supp2.pdf]

Refinement table for ENAH-PCARE crystal structure

	ENAH-PCARE (7LXF)
Wavelength	
Resolution range	45.14 - 1.65 (1.71 - 1.65)
Space group	P 61 2 2
Unit cell	52.12 52.12 197.85 90 90 120
Total reflections	742185 (53799)
Unique reflections	20193 (1951)
Multiplicity	36.8 (27.6)
Completeness (%)	99.87 (99.44)
Mean I/sigma(I)	27.78 (0.68)
Wilson B-factor	36.47
R-merge	0.0684 (3.906)
R-meas	0.0694 (3.979)
R-pim	0.0114 (0.750)
CC1/2	1 (0.659)
CC*	1 (0.891)
Reflections used in refinement	20166 (1937)
Reflections used for R-free	1012 (98)
R-work	0.217 (0.346)
R-free	0.235 (0.356)
CC(work)	0.960 (0.786)
CC(free)	0.936 (0.720)
Number of non-hydrogen atoms	1090
macromolecules	1054
solvent	36
Protein residues	134

**RMS(bonds)**	0.011
**RMS(angles)**	1.08
**Ramachandran favored (%)**	96.15
**Ramachandran allowed (%)**	3.85
**Ramachandran outliers (%)**	0.00
**Rotamer outliers (%)**	0.89
**Clashscore**	0.48
**Average B-factor**	48.68
**macromolecules**	48.85
**solvent**	43.72

Statistics for the highest-resolution shell are shown in parentheses.

Note that the $PCARE^{828\text{-}848}$ peptide is numbered as 133-153 in the PDB file, based on residue numbers in the domain-peptide fusion.
